# Low frequency blood-oxygen-level-dependent oscillations, *APOE4,* and plasma pTau_217_

**DOI:** 10.1177/13872877261467275

**Published:** 2026-07-09

**Authors:** Trevor Lohman, Arunima Kapoor, Allison C. Engstrom, Jillian Joyce, Melanie Quiring, John Paul M. Alitin, Aimee Gaubert, Amy Nguyen, Elizabeth Head, Kathleen E. Rodgers, David Bradford, Basant Lashin, S. Duke Han, Mara Mather, Daniel A. Nation

**Affiliations:** 1 Department of Neurology, 5116 University of Southern California, Keck School of Medicine, Los Angeles, CA, USA; 2 82528 University of Southern California, Leonard Davis School of Gerontology, Los Angeles, CA, USA; 3 Department of Psychological Science, 8788 University of California, Irvine, CA, USA; 4 Department of Pathology and Laboratory Medicine, 8788 University of California, Irvine, CA, USA; 5 Center for Innovations in Brain Science, Department of Pharmacology, University of Arizona, Tucson, AZ, USA; 6 Department of Psychology, 5116 University of Southern California, Los Angeles, CA, USA; 7 Department of Biomedical Engineering, 5116 University of Southern California, Los Angeles, CA, USA; 8 12223 University of Southern California, Keck School of Medicine, Los Angeles, CA, USA

**Keywords:** Alzheimer's disease, *APOE4*, BOLD oscillations, neuroimaging biomarker, pTau_217_

## Abstract

**Background:**

Low frequency oscillations in blood-oxygen-level-dependent signal (BOLD-LFOs) are generally considered nuisance signal in connectivity analysis and discarded. However, recent evidence suggests BOLD-LFOs shed light on cerebrovascular dysfunction and preclinical Alzheimer's disease, but the mechanisms remain unclear. No investigations have assessed the relationship between BOLD-LFOs and plasma pTau_217_, or how it differs in apolipoprotein ε4 (*APOE4*) carriers who are vulnerable to cerebrovascular dysfunction and genetically predisposed to AD.

**Objective:**

To study the relationship between BOLD-LFOs and plasma p-Tau_217_ in *APOE4* carriers compared to non-carriers.

**Methods:**

Independently living older adults (N = 118) were recruited and underwent resting-state fMRI and venipuncture. BOLD-LFOs were quantified as signal power within the 0.01–0.10 Hz frequency range. Plasma pTau_217_ was assessed and linear regression quantified the interactive effect of *APOE4* carrier status and BOLD-LFOs on plasma pTau_217_. 2×2 ANCOVA was used to compare BOLD-LFOs across *APOE4* carrier and amyloid positivity statuses based on previously reported pTau_217_ cutoffs.

**Results:**

The interactive effect of *APOE4* carrier status and BOLD-LFO power was significantly associated with plasma pTau_217_ (β = -0.78, p = 0.001). This relationship was driven by an inverse relationship between BOLD-LFOs and plasma pTau_217_ in *APOE4* carriers (β = −0.57, p = 0.0007). Amyloid-β (+) *APOE4* carriers displayed lower BOLD-LFOs than amyloid-β (-) *APOE4* carriers (p = 0.008) and amyloid-β (+) non-carriers (p = 0.03). Models were adjusted for age, sex, vascular risk factors, and total intracranial volume.

**Conclusions:**

Findings suggests BOLD-LFOs are implicated in preclinical AD in an *APOE4* dependent manner, adding support for the continued study of BOLD-LFOs in the context of cerebrovascular contributions to AD genetic risk.

## Introduction

Spontaneous blood-oxygen-level-dependent signal low-frequency oscillations (BOLD-LFOs) in the 0.01–0.1 Hz frequency range were once considered nuisance signal in functional magnetic resonance imaging (fMRI) connectivity analysis, caused by cardiac pulsations and respirations.^1^ However, more recent work has demonstrated that other neurophysiological processes of potential relevance to brain health contribute to BOLD-LFOs, including spontaneous cerebrovascular reactivity,^2,3^ astrocyte-mediated vasomotion,^4–6^ and neuron-astrocyte crosstalk.^7^ A growing body of evidence also indicates that there is disease-dependent variation in BOLD-LFOs,^8,9^ suggesting changes in BOLD-LFOs may have relevance to the pathophysiology of some brain diseases and could hold unique diagnostic and clinical value relative to standard fMRI.^9^

For example, widespread dampening of the amplitude of BOLD-LFOs has been observed in Alzheimer's disease (AD) dementia patients,^8^ where it correlates specifically with neuronal hypometabolism on ^18^F fluorodeoxyglucose-PET (FDG-PET). Another recent study found that BOLD-LFOs are altered early in the preclinical stages of AD, even in the absence of macrostructural atrophy.^9^ This study also found that decreased amplitude of BOLD-LFOs correlates with the degree of diffuse cerebral amyloidosis and with accumulation of tau pathophysiological change in the entorhinal cortex.^9^ Together these findings suggest that BOLD-LFO power attenuation occurs in the early stages of AD pathophysiology and is related to key amyloid, tau, and neurodegeneration markers of the disease. However, no studies to date have investigated how BOLD-LFOs relate to AD pathophysiology in the context of the AD risk gene, apolipoprotein ε4 (*APOE4*). Finally, no studies have examined whether the amplitude of BOLD-LFOs are related to blood-based biomarkers of AD, including plasma pTau_217_.

The present study aims to address this knowledge gap by investigating the relationship between BOLD-LFOs and plasma pTau_217_, a reliable diagnostic and prognostic marker of AD progression,^10^ in older *APOE4* carriers compared to non-carriers to test the hypothesis that the relationship between BOLD-LFOs and pTau_217_ differs by *APOE4* status. The present investigation also examined changes in BOLD-LFOs associated with early AD pathophysiological change by comparing BOLD-LFO power in *APOE4* carriers who have not yet developed pTau_217_ abnormality to *APOE4* carriers who have. Elevated p-Tau_217_ closely correlates with the presence of amyloid-β plaques and is associated with early amyloid changes.^10,11^ Therefore, the pTau_217_ abnormality cutoff used in the present study was based on a previous investigation identifying a single cutoff with high sensitivity and specificity to cerebral amyloidosis confirmed by cerebrospinal fluid (CSF) amyloid-β measures, enabling the comparison of BOLD-LFOs between groups categorized as either amyloid-β positive or negative.

## Methods

### Participants

Participants were recruited from Los Angeles County and Orange County communities through outreach events, mailing lists, word-of-mouth, online portals, a research volunteer registry, and through the local Alzheimer's Disease Research Center (ADRC). All procedures were conducted as part of the Vascular Senescence and Cognition (VaSC) Study at the University of Southern California (USC) and the University of California Irvine (UCI). Older adults aged 60 to 89 years who were living independently were included (Table 1). Study exclusions were a prior diagnosis of dementia, history of clinical stroke, family history of dominantly inherited neurodegenerative disorders, current neurological or major psychiatric disorders that may impact cognitive function, history of moderate-to-severe traumatic brain injury, active substance abuse, current use of medications impairing the central nervous system, current organ failure or other uncontrolled systemic illness, and contraindications for brain MRI. Eligibility for the study was verified by a structured clinical health interview and review of current medications with the participant and, when available, a knowledgeable informant study partner. This study was approved by the USC (HS-14-00784) and UCI (HS-2019-5324) Institutional Review Boards, and all participants gave informed consent in accordance with the Declaration of Helsinki. The anonymous data that support the findings of this study are available upon reasonable request from the corresponding author, DAN, through appropriate data sharing protocols.

**Table 1.** Participant characteristics and demographics grouped by ***APOE4*** carrier statu**s**.

Variable name	*APOE4* non-carriers (n = 69)	*APOE4* carriers (n = 49)	*p*
Age (years)	70.5±7.4 (60–89)	69.2±6.7 (60–89)	0.30^a^
Sex (n, female %)	49 (71.0)	31 (63.3)	0.42^b^
BOLD-LFOs (0.01–0.1 Hz power)	98.82±81.1	103.7±93.1	0.77^a^
Plasma pTau_217_ (pg/ml)	0.30±0.17	0.42±.27	0.01^a^
Amyloid-β status^c^ (n, amyloid+ %)	12 (17.4)	17 (34.7)	0.04^b^
≥2 Vascular Risk Factors (n, %)	36 (52)	24 (49)	0.85 ^b^
*APOE2/3* heterozygotes (n, %)	2 (3)	-	-
*APOE3/3* homozygotes (n, %)	67 (97)	-	-
*APOE2/4* heterozygotes (n, %)	-	2 (4)	-
*APOE3/4* heterozygotes (n, %)	-	45 (92)	-
*APOE4/4* homozygotes (n, %)	-	2 (4)	-

*APOE4:* apolipoprotein ε4 allele; BOLD-LFO: gray matter blood oxygen level dependent low frequency oscillations (0.01–0.1 Hz). ^a^independent t-test, ^b^chi-squared test, ^c^amyloid-β status determined based on previously reported pTau_217_ cutoff of 0.44 pg/ml which displays high combination sensitivity/specificity for detecting cerebral amyloidosis based on cerebrospinal fluid Aβ_42/40_.^14^

### Neuroimaging

All participants underwent brain MRI scans conducted on a 3T Siemens Prisma scanner with 20-channel head coil. High-resolution 3D T1-weighted anatomical (Scan parameters: TR = 2300 ms; TE = 2.98 ms; TI = 900 ms; flip angle = 9 deg; FOV = 256 mm; resolution = 1.0 × 1.0 × 1.2 mm3; Scan time = 9 min) images were acquired, using 3-dimensional magnetization-prepared rapid gradient-echo (MPRAGE) sequences. Resting state fMRI scans comprised 140 contiguous echo-planar imaging (EPI) functional volumes (TR = 3000 ms, TE = 30 ms, FA = 80°, 3.3 × 3.3 × 3.3 mm voxels, matrix = 64 × 64, FoV = 212 mm, 48 slices).

Resting-state functional MRI data were preprocessed using a custom Python pipeline that integrates FMRIB Software Library (FSL v6.0), NiBabel, and SciPy. Preprocessing was conducted on each participant's BOLD image series and corresponding T1-weighted anatomical scan.

Each participant's T1-weighted anatomical image was skull-stripped using FSL's BET and segmented into gray matter and white matter probability maps using the FAST tool. These maps were linearly registered to the participant's functional space using FLIRT,^12^ and binary tissue masks were generated by thresholding the probabilistic segmentations at 0.5. The fully preprocessed functional images were then registered to MNI152 standard space (2 mm resolution) using affine registration with 12 degrees of freedom.

The first 10 volumes of each BOLD time series were discarded. Slice timing correction was applied to adjust for inter-slice acquisition delays, as well as motion correction using FSL's MCFLIRT tool to realign all volumes to reference image. After realignment, linear trends were removed from each voxel's time series using a Python-based detrending procedure implemented with SciPy. The detrended data were spatially smoothed with a Gaussian kernel (4 mm full width at half maximum, approximated by sigma = 1.7 voxels) and temporally band-pass filtered to extract frequencies between 0.01–0.10 Hz using FSL's fslmaths command.

Mean BOLD time series were extracted from the filtered functional images using the previously created gray matter mask. For each extracted time series, power spectral density (PSD) was calculated via fast Fourier transform, and total power within the 0.01–0.10 Hz frequency band was computed.

Routine nuisance regression of global signal, CSF signal, and motion parameters was not performed in order to avoid loss of hemodynamic information, as demonstrated elsewhere.^12,13^

### Plasma AD biomarkers

pTau_217_ concentrations were quantified in human plasma samples using the ultra-sensitive Simoa (Single Molecule Array) assay platform developed by Quanterix (Lexington, MA, USA). The pTau_217_ assay utilized a bead-based sandwich 3 step immunoassay format run on the Quanterix HD-X Analyzer, which allows for digital quantification of low-abundance biomarkers in biological fluids. Plasma samples were collected in K2EDTA tubes, centrifuged at 2000 x g for 10 min at 4°C, and stored at −80°C until analysis. Prior to assay, samples were thawed on ice and centrifuged again to remove any debris. The pTau_217_ assay was performed according to the manufacturer's instructions (Quanterix pTau_217_ Advantage Kit, Catalog # 104588), using 100 μL of plasma per well. Capture antibodies specific for pTau_217_ were immobilized on paramagnetic beads, while detector antibodies were labeled with a proprietary enzyme tag. Following incubation and washing steps, individual immunocomplexes were transferred to a microwell array, allowing for single-molecule detection. A chemiluminescent substrate was added, and signals were captured digitally by the HD-X Analyzer.

A plasma pTau_217_ cut off of 0.44 pg/ml has previously displayed high combination specificity and sensitivity (>85%) for detecting cerebral amyloidosis based on CSF Aβ_42/40._^14^This cutoff was used in the present study to categorize participants as either amyloid-β positive or negative.

### APOE genotyping

Fasted blood samples were obtained by venipuncture and used to determine participant *APOE* genotype. Genomic DNA was extracted using the PureLink Genomic DNA Mini Kit (Thermo). *APOE* genotyping was performed as previously described.^15–17^ *APOE4* carriers were defined as participants with at least one copy of the *APOE* ε4 allele. All analyses were performed at the same lab at the University of Arizona (KR).

### Vascular risk factors

Vascular risk factor (VRF) burden was determined through clinical interviews with the participant and informant (when available), and review of current medications and medical history. The assessed VRFs included history of cardiovascular disease (e.g., heart failure, angina, stent placement, coronary artery bypass graft, intermittent claudication), hypertension, hyperlipidemia, type 2 diabetes, atrial fibrillation, left ventricular hypertrophy, current smoking status, and transient ischemic attack. Hypertension was operationally defined as self-reported diagnosis of hypertension or taking antihypertensive medications. Type 1 diabetics were excluded, and type II diabetes was operationally defined as self-reported diagnosis at clinical interview. Total VRFs were summed for each participant and elevated VRF burden was defined as ≥2 VRFs (versus 0–1) as described previously.^18,19^

### Statistical analyses

121 participants underwent brain fMRI, anatomical T1w MRI, and venipuncture. All data were screened for outliers (±3 SD) and three gray matter BOLD-LFO outliers were identified and removed with values of +4.84 SD, +4.22 SD, and +3.79 SD. After outlier screen, the total analyzed sample was N = 118. All assessed variables were compared between *APOE4* carriers and non-carriers using independent t-tests.

Data analyses were performed in R. Hayes PROCESS Model 1 (simple moderation) was used to assess the potential moderating effect of *APOE4* carrier status on the relationship between BOLD-LFOs and plasma pTau_217_ (primary analysis), where x = BOLD-LFO, Y = pTau_217_, and w = *APOE4* carrier status. Linear regression assumptions regarding linearity, multicollinearity (VIF<5), and homoscedasticity (Breusch-Pagan test) were met. Lastly, within group analyses were performed to assess the relationship between BOLD-LFOs and pTau_217_ within the *APOE4* carrier and non-carrier groups. A second multivariate model was ran adjusted for age, sex, vascular risk factor burden, and intracranial volume. Additional sensitivity analysis of the interactive effect was tested with *APOE2* carriers (n = 4) excluded.

2X2 ANCOVA was used to compare BOLD-LFOs by amyloid-β positivity and *APOE4* carrier status and pairwise comparisons were performed (secondary analysis). A second 2X2 ANCOVA was ran adjusted for age, sex, vascular risk factor burden, and intracranial volume.

For illustration purposes, power spectral density plots were generated to compare BOLD-LFOs power between amyloid-β (+) and amyloid-β (-) *APOE4* carriers. A power spectral density plot is a frequency-domain graph that shows how the power of a signal is distributed across a frequency range, in this case, 0 to 0.15 Hz which represents the frequency band captured by a 3000 ms TR during fMRI.

Minimum detectable effect sizes given a power of 0.80, alpha of 0.05, two covariates, and a sample size of N = 118 was calculated using the pwr package in R as ΔR^2^ = 0.05 for the BOLD-LFO*APOE4 interaction effect. False discovery rate correction was used to account for multiple comparisons in the primary analysis including APOE4*BOLD-LFOs interaction effect and subgroup analyses (within *APOE4* carrier and non-carrier group effects).^20^

## Results

118 participants were included for analysis, participant characteristics and demographics for this sample are shown in Table 1 grouped by *APOE4* carrier status.

**Figure 1.**
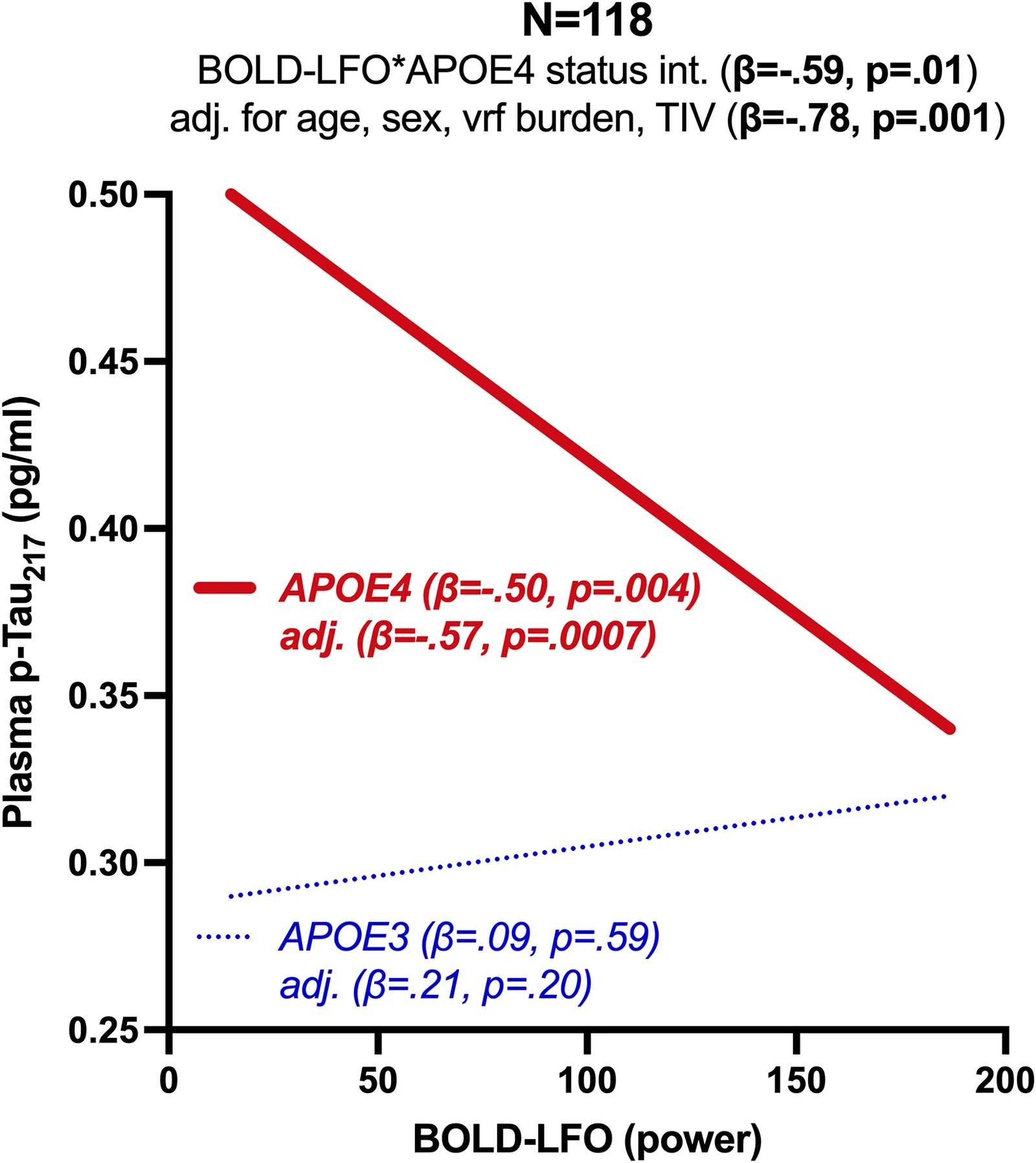
The relationship between 0.01–0.1 Hz gray matter BOLD low frequency oscillations (BOLD-LFO) and pTau_217_ in *APOE4* carriers (n = 49) compared to *APOE3* homozygotes (n = 69). The effect of BOLD-LFOs on pTau_217_ conditional upon *APOE4* carrier status (interaction effect) is reported as standardized regression coefficient (*β*) and p-value in the total sample (N = 118) with and without covariate adjustment (age, sex, vascular risk factor (vrf) burden, and total intracranial volume (TIV)). Within group statistics reported in figure legend as *β* and p-value within *APOE4* carrier status groups with and without covariate adjustment.

The interactive effect of *APOE4* carrier status and gray matter BOLD-LFOs was significantly associated with plasma pTau_217_ in the univariate analysis (β = −0.59, 95% CI (−1.06, −0.12), p = 0.01) and also after adjustment for age, sex, vascular risk factor burden, and total intracranial volume (β = −0.78, 95% CI (−1.24, −0.32), p = 0.001 Table 2). This was driven by an inverse relationship between BOLD-LFOs and pTau_217_ in *APOE4* carriers (within-group) as shown in Figure 1.

**Table 2.** Regression coefficients for model predicting plasma pTau_217_.

Variable name	β	95% CI	*p*
BOLD-LFOs (0.01–0.1 Hz power)	0.21	(−0.11, 0.53)	0.20
Sex (female)	0.04	(−0.53, 0.44)	0.86
Age	0.35	(0.18, 0.53)	**0.0001**
*APOE4*	0.51	(0.16, 0.86)	**0.005**
≥2 Vascular Risk Factors	−0.22	(−0.58, 0.13)	0.22
Total Intracranial Volume	0.21	(−0.003, 0.43)	0.05
BOLD-LFO*APOE4	−0.78	(−1.24, −0.32)	**0.001**

*APOE4:* apolipoprotein ε4 allele; β: standardized beta coefficient; BOLD-LFO: gray matter blood oxygen level dependent low frequency oscillations (0.01–0.1 Hz).

**Figure 2.**
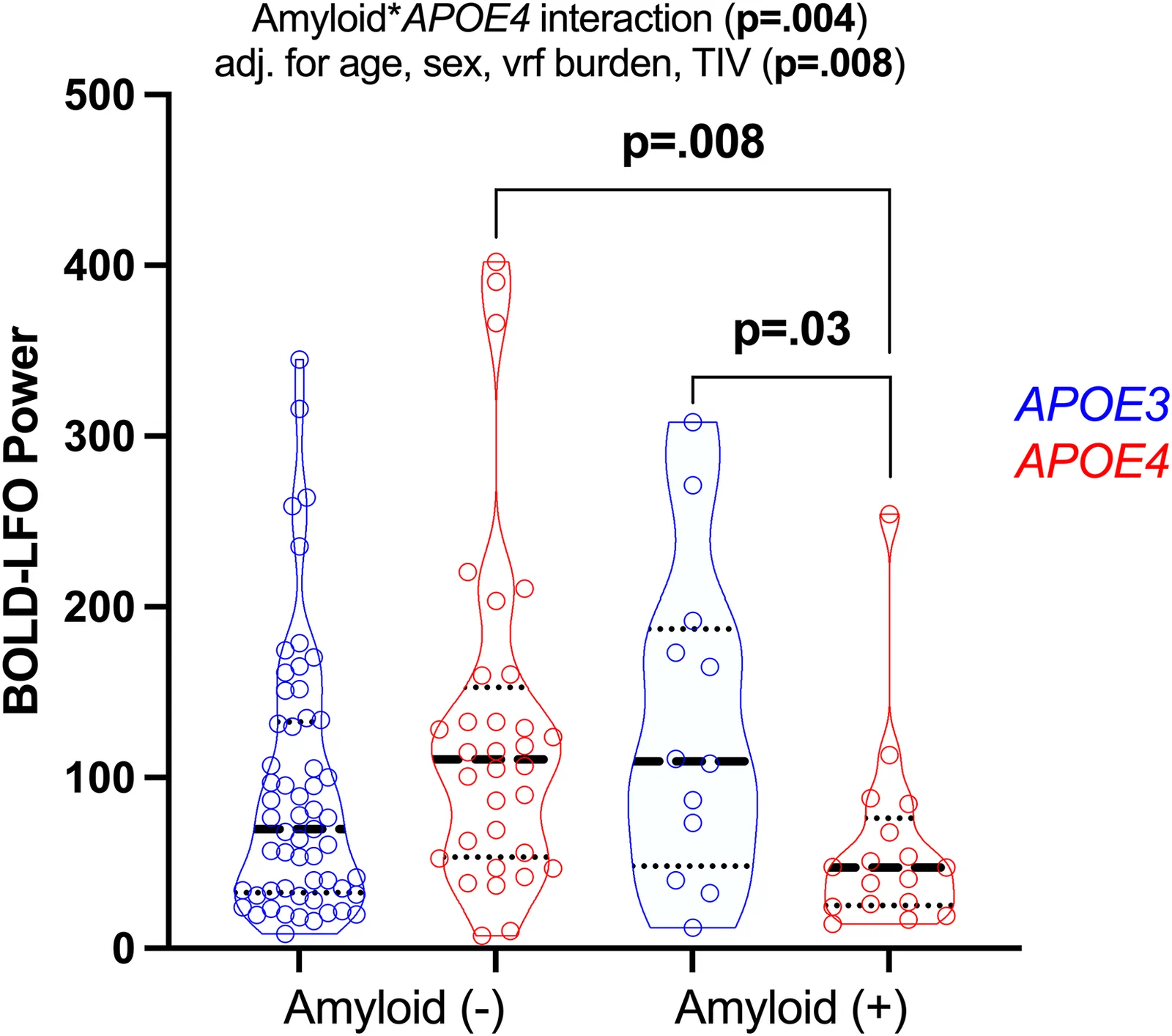
Gray matter blood oxygen level dependent low frequency 0.01–0.10 Hz oscillations (BOLD-LFO) comparisons of *APOE4* status (*APOE3* homozygotes versus *APOE4* carriers) by amyloid-β positivity status. 2X2 ANCOVA interaction effect p-value in the total sample (N = 118) is reported with and without covariate adjustment (age, sex, vascular risk factor (vrf) burden, and total intracranial volume (TIV)). Pairwise comparisons were conducted using unadjusted t-tests of group means. Amyloid-β status determined based on previously established pTau_217_ cutoff of 0.44 pg/ml which displays high combination sensitivity/specificity for detecting cerebral amyloidosis based on cerebrospinal fluid Aβ_42/40_.^14^.

To visualize this effect across pTau_217_ cutoffs and *APOE4* carrier status groups, a 2X2 ANCOVA with pairwise comparisons was performed. The amyloid-β positivity status*APOE4 carrier status interaction was statistically significant (β = −0.91, 95% CI (−1.53, −0.29), p = 0.004), and remained so after adjustment for age, sex, vascular risk factor burden, and total intracranial volume, (β = −0.95, 95% CI (−1.64, −0.25), p = 0.008). In pairwise comparisons amyloid-β positive *APOE4* carriers displayed lower BOLD-LFO power than amyloid-β positive *APOE3* homozygotes (Δz = 0.61, 95% CI (0.08, 1.14), p = 0.03), and amyloid-β negative *APOE4* carriers (Δz = 0.58, 95% CI (0.15, 1.00), p = 0.008) (Figure 2).

**Figure 3.**
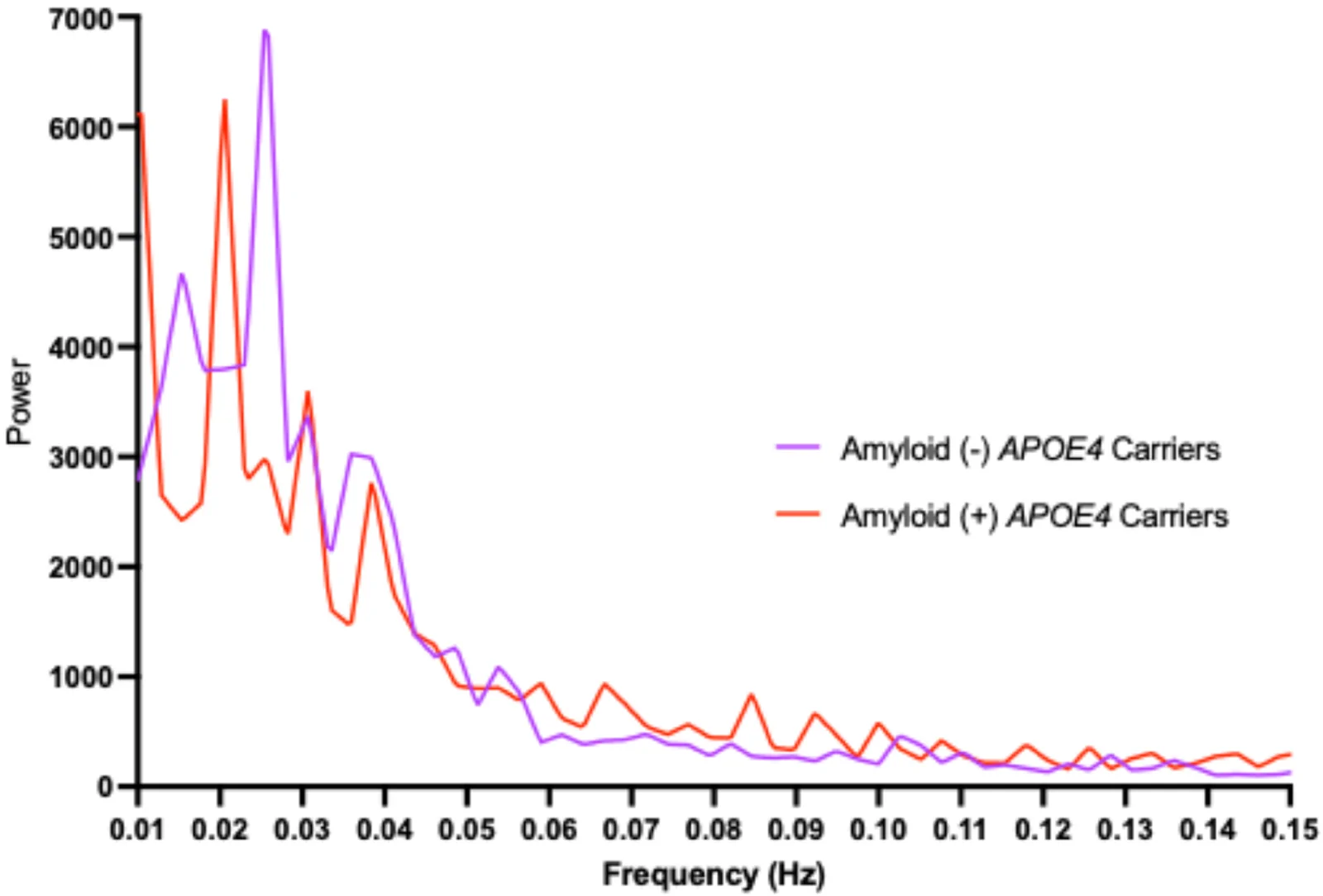
Gray matter blood oxygen level dependent low frequency oscillations (BOLD-LFO) power spectral density plot. Comparisons across amyloid-β positivity status (amyloid-β positive =  pTau_217_>0.44 pg/ml) in *APOE4* carriers. Amyloid-β positive n = 17, amyloid-β negative n = 32. Amyloid-β status determined based on previously reported pTau217 cutoff of 0.44 pg/ml which displays high combination sensitivity/specificity for detecting cerebral amyloidosis based on cerebrospinal fluid Aβ_42/40._^14^.

A power spectral density plot is shown in Figure 3 to visualize the BOLD-LFO differences between amyloid-β positive and negative *APOE4* carriers across the full frequency spectrum collected during fMRI. This graph is for illustrative purposes to visualize which frequency bands drive the previous finding that amyloid-β positivity status is related to BOLD-LFO power (<0.1 Hz) in *APOE4* carriers. This finding appears to be driven by decreased power in the <0.05Hz frequency range as shown in Figure 3.

### Sensitivity analyses and multiple comparisons

To account for potential residual confounding the interactive effect of *APOE4* carrier status and gray matter BOLD-LFOs on plasma pTau_217_ was tested after excluding participants with a copy of the *APOE2* allele (n = 4) which did not attenuate the interactive effect of *APOE4* carrier status and gray matter BOLD-LFOs on plasma pTau217 (β = −0.82, 95% CI (−1.32, −0.31) p = 0.002). The BOLD-LFO*APOE4 interaction term effects and subsequent within group analysis results survived correction for multiple comparisons.

## Discussion

The present study finds that BOLD-LFO power is inversely associated with plasma pTau_217_ levels in *APOE4* carriers but not in non-carriers, suggesting a role for the *APOE4* gene in modifying the relationship between BOLD-LFOs and AD pathophysiological change. Findings also suggest this alteration in BOLD-LFOs may be observed in preclinical AD, as evidenced by increased BOLD-LFO power in amyloid-β negative *APOE4* carriers compared to amyloid-β positive *APOE4* carriers. Together these results are consistent with prior work suggesting changes in BOLD-LFOs are related to AD pathophysiological changes measured by PET and CSF biomarkers^9^ and extend prior findings by demonstrating the relationship between decreased BOLD-LFOs and plasma pTau_217_ in *APOE4* carriers specifically.

*APOE4* is associated with vascular, neuroinflammatory, and metabolic changes in neurons^21,22^ and neuroglia.^23–26^ One or more of these neurophysiological changes could be implicated in the relationship between BOLD-LFOs and pTau_217_ in *APOE4* carriers. For example, *APOE4* conveys susceptibility to cerebrovascular dysfunction,^27–29^ which could contribute to the observed decreased in BOLD-LFOs through decreased pulsatile or intrinsic vasomotion.^2,3^ Astrocytes are also increasingly thought to be important contributors to BOLD-LFO signal^7,30^ and *APOE4* is associated with numerous changes to astrocyte function caused by an accumulation of lipid droplets and a buildup of unsaturated fatty acids within astrocytes.^23^ This accumulation of lipid droplets is sufficient to induce astrocyte reactivity, triggering the secretion of inflammatory chemokines and cytokines.^31,32^

An examination of BOLD-LFO differences in amyloid-β positive and amyloid-β negative *APOE4* carriers in the present study suggests that the largest differences in power may exist in the lower frequency ranges, which are associated with astrocyte-mediated vasomotion,^4,5,7,30,33^ particularly vasodilation.^6^ Thus, the observed relationship between reduced BOLD-LFOs and AD pathophysiology in APOE4 carriers may be related to changes in vascular function, astrocyte reactivity, or astrocyte-vascular interactions.^34^ However, other systemic physiological processes like cardiac pulsations and respiratory dynamics contribute to BOLD-LFO signal, and further studies are needed to determine the specific mechanistic contributions to BOLD-LFOs that may be implicated in *APOE4* associated AD pathophysiology.

This study highlights the relationship between cerebral hemodynamics and preclinical AD pathophysiology. Future studies should seek to determine if BOLD-LFOs represent a causal contribution to amyloid-β or tau pathology, or if the relationship is purely associative. If BOLD-LFOs represent a distinct mechanistic contribution to AD pathophysiology this would have significant implications for the study of cerebrovascular function in the context of AD. If instead, they are merely associated with early AD pathophysiological changes in *APOE4* carriers then they may represent a novel imaging biomarker of AD pathophysiology. Strengths of the present investigation include the study of BOLD-LFOs in preclinical AD by comparing *APOE4* carriers to non-carriers with and without pTau_217_ abnormality. Limitations include the associative, cross-sectional nature of the study and the inference of cerebral amyloidosis status using a plasma pTau_217_ cutoff rather than measuring it directly using Aβ PET, plasma, or CSF assay. The present study findings suggest that BOLD-LFO changes may be implicated in preclinical AD, particularly in *APOE4* carriers.
